# Influence of Acid Swallows on the Dynamics of the Upper Esophageal Sphincter

**DOI:** 10.1007/s00455-020-10159-2

**Published:** 2020-09-04

**Authors:** Simone Miller, Martin Ptok, Michael Jungheim

**Affiliations:** Department of Phoniatrics and Pediatric Audiology, Hannover Medical School, Carl-Neuberg-Straße 1, Hannover, Germany

**Keywords:** Acid, Upper esophageal sphincter, Restitution time, High-resolution manometry, Swallow, Deglutition

## Abstract

Mechanisms of the upper esophageal sphincter (UES) when exposed to acid are still incompletely understood. The presented work investigated the reaction of the UES to acid exposure during swallowing. Ten healthy individuals swallowed ten 2 ml neutral water boli of pH 7, followed by 10 swallows each of different levels of acidity (pH 1.8, pH 3 and pH 5). Effects were analyzed by high-resolution manometry (HRM) for the primary parameter Restitution Time, as well as Resting Pressures, maximal, minimal pressures and time intervals. Restitution Times measured mean values of 12.67 s (SD ± 7.03 s) for pH 1.8, pH 7 = 8.69 s (SD ± 2.72 s), pH 3 = 7.56 s (SD ± 2.23 s) and pH 5 = 7.29 s (SD ± 2.55 s), showing prolonged Restitution Times in the UES when exposed to strong bolus acidity. This difference was significant towards the neutral bolus, but also to less acidic boli (pH 5: *p* = 0.006, pH 3: *p* = 0.009, pH 7: *p* = 0.038). Considerable differences of mean values were found for Post-Swallow Maximum and Period of Sphincter Activity. Also, Pre-Swallow Maximum values were found to be highest with the strongest acid. Relaxation Times showed a slight trend of prolongation for the highest bolus acidity. Prolonged Restitution Times may represent a reflexive protective mechanism triggered by receptors in the pharyngeal mucosa or the UES preventing regurgitation of acid into the pharynx and larynx, besides representing ongoing attempts of acid clearance. Exposure to high levels of acidity by a swallowed bolus does influence UES functions during swallowing.

## Background and Aims

The upper esophageal sphincter (UES) denotes the pharyngo-esophageal segment. It presents with permanent pressure during its state of rest in order to prevent regurgitation of material into the pharynx and air ingestion into the esophagus [1]. Apart from this, it has an important function during swallowing—as it needs to relax and open in order to let the bolus pass—and is postulated to act as a barrier to prevent pharyngolaryngeal reflux [2]. Sphincter pressures are influenced by a number of factors and may vary in relation with the state of arousal, state of awareness (sleep vs. awake), activities or respiration among others [3–8].

As many influencing factors have already been investigated, the mechanism of the UES when exposed to acid during food intake is still uncertain. Some authors have observed an increase in sphincter pressure due to acid exposure of the esophagus [9–14], whereas others did not find any changes in UES pressure when exposed experimentally or spontaneously [5, 15, 16]. Most of these studies have investigated spontaneous reflux events or experimental infusion with hydrochloric acid (HCl) into the esophagus in order to mimic reflux events. One study has investigated sensory stimulation in terms of swallowed boli, but did not report of significant pressure changes in the UES in relation with a slightly sour bolus [14]. Following reports on missing UES reactions in patients with reflux [17], it has even been proposed that the UES may not take part in preventing esophagopharyngeal reflux at all [18].

Considering further factors influencing sphincter pressures, it has been suggested, that apart from, or even instead of acidity, factors such as volume [12], distension [19, 20] infusion rate [12] and distance to the UES [12, 19, 20] affect the UES tone and/or activity. Thompson et al. [20] even concluded that the distension of the esophagus due to perfusion causes UES reactions, as in pressure changes, rather than the pH-concentration of solutions. Similarly, it has been reported that reflux-induced intraesophageal pressure increases caused an increase in UES pressure, whether reflux events were acidic or non-acidic [10]. Similarly as for UES activity it was said that distension of the proximal esophagus as well as the distal acid perfusion are influencing factors [21]. It becomes apparent that not enough evidence has been provided with regard to the different theories concerning the influences of acidic exposure of the esophagus and UES.

Despite the studies implemented, mechanisms of the UES when exposed to acid are still incompletely understood. So far the focus has been placed on acid reflux or methods resembling reflux disease. This study aims to investigate the reaction of the UES to different levels of acid exposure not by infusion into the esophagus, but during swallowing. It shall be determined, whether the exposure to acid does influence swallowing pressures and pharyngeal motor functions, reflexive activities and whether or not these effects change with acid concentration. Effects of acid exposure are investigated during the pharyngeal phase of swallowing, by swallowed boli of different levels of acidity.

## Methods

This study was conducted after approval of the Committee of Ethics in Research of the Hannover Medical School (no. 6222, 2012) and consent from all participants.

### Participants

Ten healthy volunteers, between 22 and 52 years of age (average age: 35.4 years), five males and five females participated in this study. All subjects met the following inclusion criteria: consent, absence of diseases of the esophagus, absence of any symptoms of dysphagia, not pregnant, no previous operations of the throat or esophagus, no diagnosed reflux disease or use of proton pump inhibitors.

### High-Resolution Manometry

A solid-state, high-resolution manometry system (Solar GI HRM, Medical Measurement Systems (MMS), and software MMS Database V8.20, Enschede, The Netherlands) and a manometric catheter (Unisensor, Attikon, Switzerland) were used for data collection. This catheter has especially been designed to measure swallowing pressures in the pharynx and UES. The flexible manometric catheter had an outer diameter of 2 mm (6 French) and a total of 20 unidirectional pressure sensors. 19 sensors measure 0.75 cm apart and one further distal sensor is placed 5 cm apart in order to monitor esophageal pressures. Measurements were recorded at a frequency of 50 Hz.

### pH-Meter

A digital pH-value measuring device (PH100 ATC, Voltcraft, Hirschau) with a resolution of 0.01 pH and automatic temperature compensation was used to ensure exact levels of acidity for the experimental boli. It detects pH-values between 0.0 and 14.0 with an accuracy of ± 0.1 pH (pH 4–10), ± 0.07 pH (pH 5–9), ± 0.2 pH (pH 1–4). The system was calibrated using a two-point calibration (7.00 and 4.00 pH-buffer/calibration solution).

### Data Collection

The manometric catheter was placed transnasally into the proximal esophagus. The high-pressure area of the UES was visible in the software and enabled placement of the probe in a way that the pharynx and proximal esophagus were indicated. Once the catheter was placed correctly, it was fixed in place at the tip of the nose and participants sat quietly for ten minutes to adjust to the situation before performing experimental swallows. To avoid a loss of sensitivity of the mucosa, no lubricating gel containing local anesthetics was used.

Each individual swallowed 2 ml boli of pH-neutral and acidic water at room temperature delivered into the oral cavity by syringe and swallowed on command of the examiner. Syringes were filled by the investigator, in order to minimize muscle activities influencing sphincter pressure. Four series of tests were performed: The individuals started by swallowing 2 ml boli of pH 7 ten times, which served as reference values. These neutral swallows were followed by ten swallows of each pH 1.8, pH 3 and pH 5. The sour boli were created by mixing citric acid (E 330, Dr. August Oetker Nahrungsmittel KG, Bielefeld, Germany for use in foodstuffs) and water. Each acid bolus was followed by a neutralizing water bolus. Bolus intakes were separated by at least 30 s in order for the swallowing activity to have ceased before a new test swallow occurred [21]. Swallows were performed sitting upright with the head in a neutral position (facing forward). In between test settings (series of pH 1.8; pH 3 and pH 5) patients waited for 15 min and drank some water in order to neutralize the oral and pharyngeal cavities.

A bolus of 2 ml was chosen in order to enable swallowing, but at the same time to not modify swallowing parameters. It was aimed to resemble natural swallowing episodes of saliva.

### Parameter Definition

In order to assess the influences of acid exposure onto the pharyngo-esophageal segment, specific parameters were extracted from the collected data [22, 23].

## Primary Parameter: Restitution Time (*t*_resti_)

**Fig. 1:**
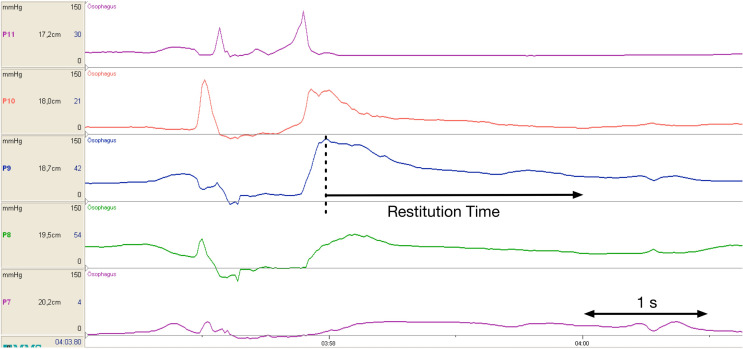
The time interval of restitution (*t*_resti_) stretching from the arrival of the peristaltic wave in the upper esophageal sphincter (UES) until the UES reaches its Resting Pressure again (x-axis = time, y-axis = pressures per sensor (P) in mmHg)

The period of restitution describes the time interval from the arrival of the peristaltic wave in the UES until the UES reaches its resting pressure again (Fig. 1). The peristaltic wave is seen as a high-pressure area moving caudally from the velum to the UES in order to move the food bolus. The UES contracts more strongly after the bolus has passed through [24], before it eventually returns to its state of rest (Resting Pressure).

**Fig. 2:**
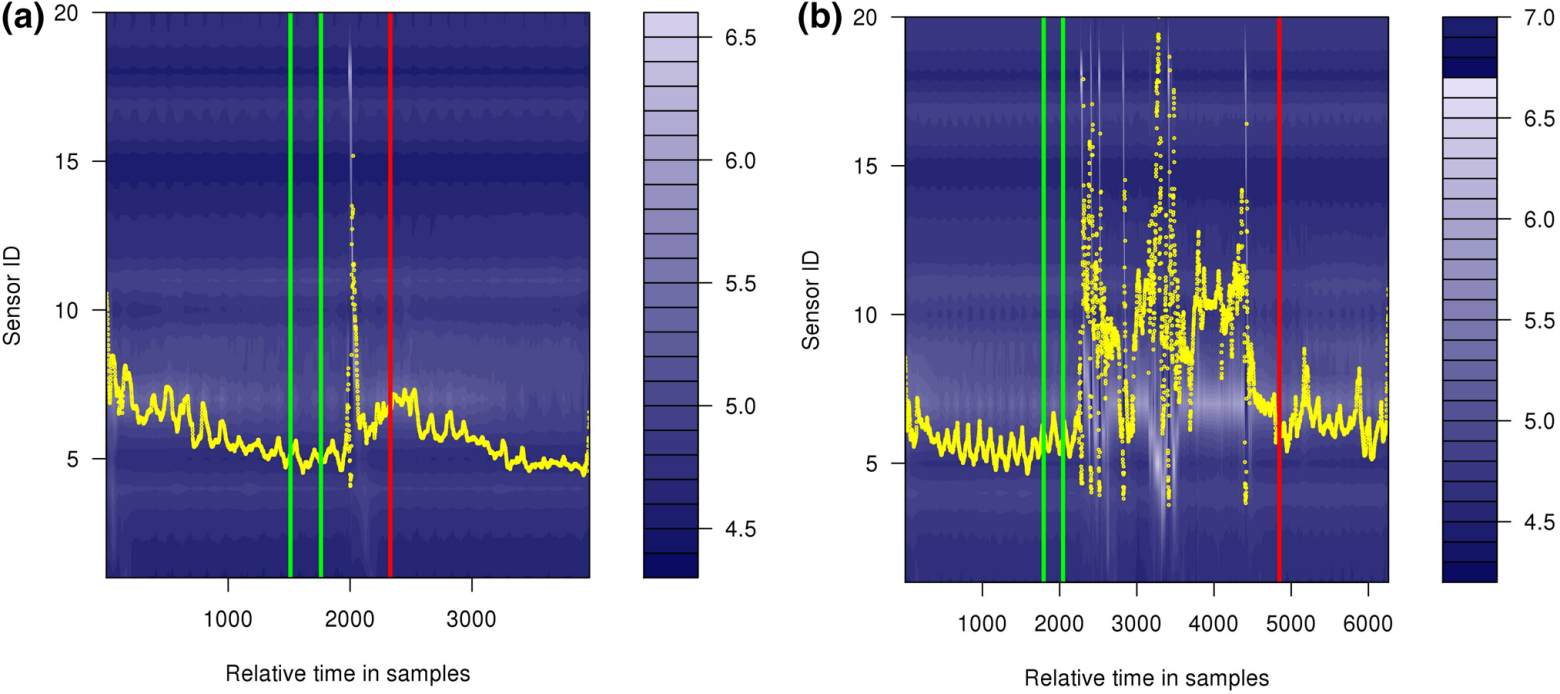
Model-based prediction of Restitution Times (indicated by the red line) in a swallow of a neutral bolus (**a** pH 7) [38] and an acid bolus (**b** pH 1.8). The green lines indicate the time interval of the Resting Pressure. The maximum pressure curve is shown in yellow. The *x*-axis shows a relative time in samples and the *y*-axis as a multi axis denotes the pressure sensors on the catheter (P1–P20) as well as (scaled) pressures in mmHg. Individual sensor pressures are color coded and shown as a contour plot (see bar on the right) [38]

In order to determine Restitution Times more objectively, machine learning has been used and an automated model has been developed (previously described [25, 26]). It uses a sequence labeling approach for labeling all time samples of a swallow to determine, whether or not swallow-related activities are present at a given point in time. Based on the assumption that a single swallow ideally consists of two state transitions, the first being the transition from the ‘non-swallowing’ to a ‘swallowing’ state and the second representing the transition back from the ‘swallowing’ to the ‘non-swallowing’ state, the model infers the Restitution Time (*t*_resti_) based on the first occurrence of the latter transition (Fig. 2). In order to cope with the large amount of data and measurement dependent variations, post-preprocessing techniques such as posterior smoothing were employed to average out jittering effects.

**Fig. 3:**
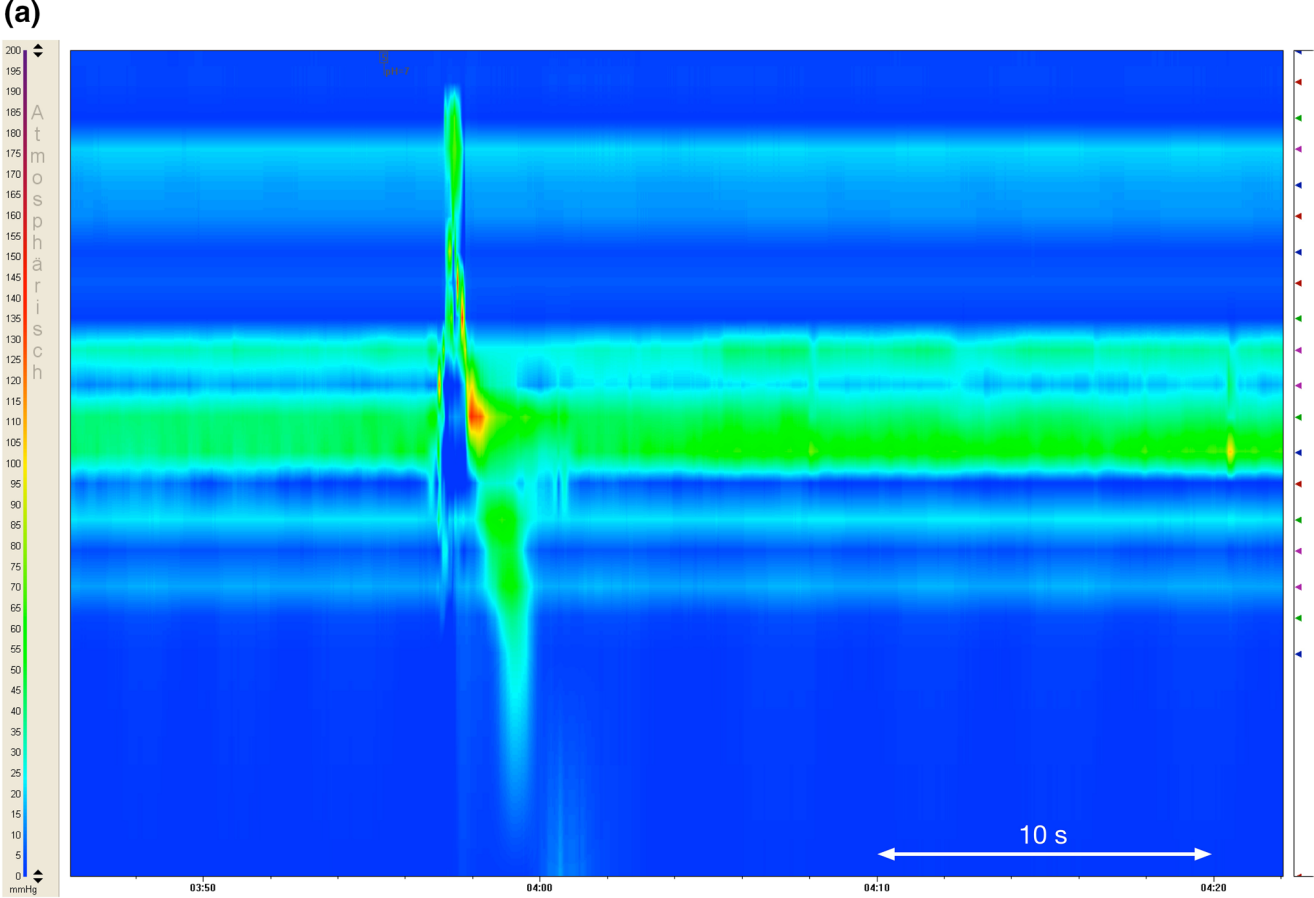

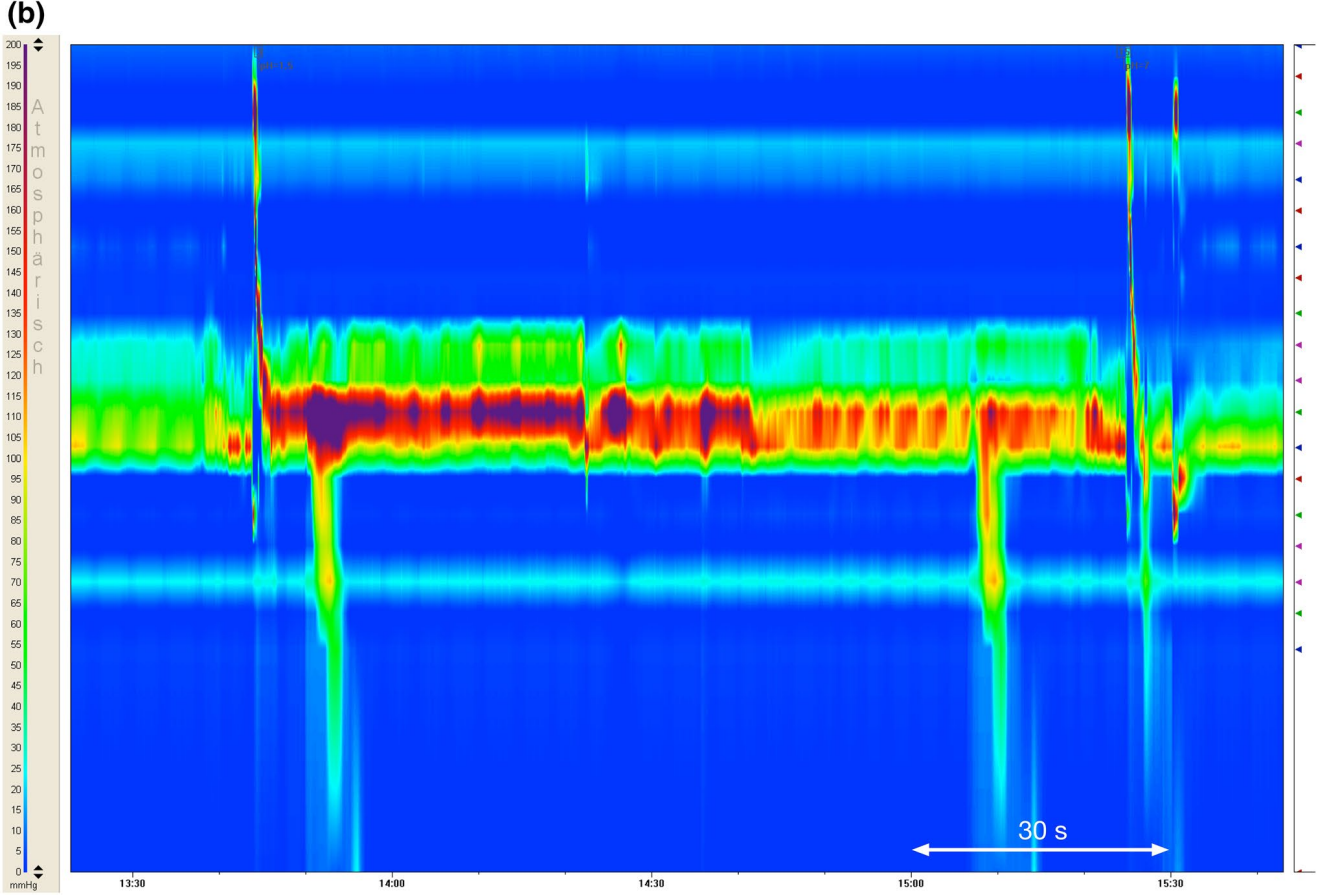
**a** pH-neutral swallow showing restitution patterns [38]. (*x* = time, *y* = penetration depth of the catheter, pressures are color coded from blue = 0 to purple = 200 mmHg). **b** Patterns of restitution in relation with acid swallows (pH 1.8), showing existing “Oscillations” as a prolonged presence of very high pressures

During Restitution Times, one sub-parameter, named Oscillations, was determined manually. Oscillations describe obvious—as in visual detection and classification—prolonged or repetitive periods of high-pressure intervals of variable frequency and duration in the UES during the phase of restitution as shown in Fig. 3b (in contrast to no Oscillation as in Fig. 3a).

## Secondary Parameters

### Average Values

#### Resting Pressure

The Resting Pressure was averaged over a 5 s period and the sensors were defined to lie within the UES before the onset of any swallowing related activity. The placement of the window was corrected manually if necessary.

### Minimal Values

#### Residual Pressure

The Residual Pressure represents the lowest recorded pressure during the period of relaxation within the area of the upper esophageal sphincter.

### Maximal Values

#### Pre-Swallow Maximum (pre-max)

The Pre-Swallow Maximum describes the highest pressure recorded in the Region of Interest (ROI) of the UES, stretching across four sensors above and one sensor below the sensor representing UES (modified from [22]), just prior to the relaxation. In order to confine the Pre-Swallow Maximum from sphincter activity related to water being delivered into the oral cavity via syringe, only maximum values within a time frame of 500 ms prior to the onset of velopharyngeal activity were considered.

#### Post-Swallow Maximum (post-max)

The Post-Swallow Maximum represents the highest pressure recorded after the arrival of the peristaltic wave and as part of an initial contractive response within area of the UES, stretching across four sensors cranial to the sensor representing UES, to allow for the swallow-related movement of the sphincter (in accordance with [22]).

### Time Intervals

#### Relaxation Time (*t*_relax_)

The onset of the Relaxation Time was defined as a 10% pressure drop from the Resting Pressure and the offset as the point when the same pressure value was reached again due to the arrival of the peristaltic wave (see [16, 27, 28]). Pressures were recorded from the central sensor in the UES. The central sensor was chosen in order to make values comparable, as the time window narrows in cranial and widens in caudal direction.

#### Period of Sphincter Activity (*t*_UESact_)

The Period of Sphincter Activity describes the time window between pre-max and post-max (see [22]).

### Pilot Study

Beforehand, a pilot study has investigated whether or not sphincter activity during acid exposure showed any deviations from pH-neutral swallows. Three healthy volunteers (average age: 29.7 years), one male and two females, were instructed to swallow 2 ml boli of two different pH-concentrations. Subjects swallowed pH-neutral, pH 1.8 and again pH-neutral boli five times each. In between different pH-consistencies (modalities), hence after five swallows, the subjects rested for 15 min in order to neutralize the structures involved.

Pressures in the pharynx and upper esophagus were recorded by means of High-Resolution Manometry (HRM). Resting Pressures, Relaxation Times, Restitution Times and post-max pressures were calculated and mean values assessed per person and modality. The Restitution Time seemed to cover effects of acid on swallowing best and was chosen as the primary outcome parameter. Mean Restitution Times were assessed per person and modality. Considering type I error (95%) and power (80%), a sample size of 8 subjects was determined. The small amount of subjects necessary resulted from the large effect size. The parameter Restitution Time was then investigated for significance in this study.

### Statistical Analysis

Data were analyzed using SPSS (Version 22.0). Each subject included in the study was included in the analysis. Restitution Time denotes the primary endpoint. The effect size for Restitution Times and sample sizes have been determined based on the effect size shown during the previously implemented pilot study.

Mean values were established per person and modality across the ten swallows (aggregated values) in order to ensure high accuracy. A one-way analysis of variance was carried out at first to check for differences between the four different groups. If this test reached statistical significance, further pairwise comparisons were carried out.

The categorical sub-parameter “Oscillations” was analyzed using a cross-classified table and Pearson’s Chi-Square Test.

## Results

The trials were all implemented with pH-neutral control swallows as the first test series. Even though subjects only started experimental swallows after an adjustment period of ten minutes after the catheter had been placed, it has previously been shown that UES Resting Pressures are higher at the beginning of trials than towards the end [28], as a continuing adjustment to the catheter is taking place.

### Restitution Times (*t*_resti_)

**Fig. 4:**
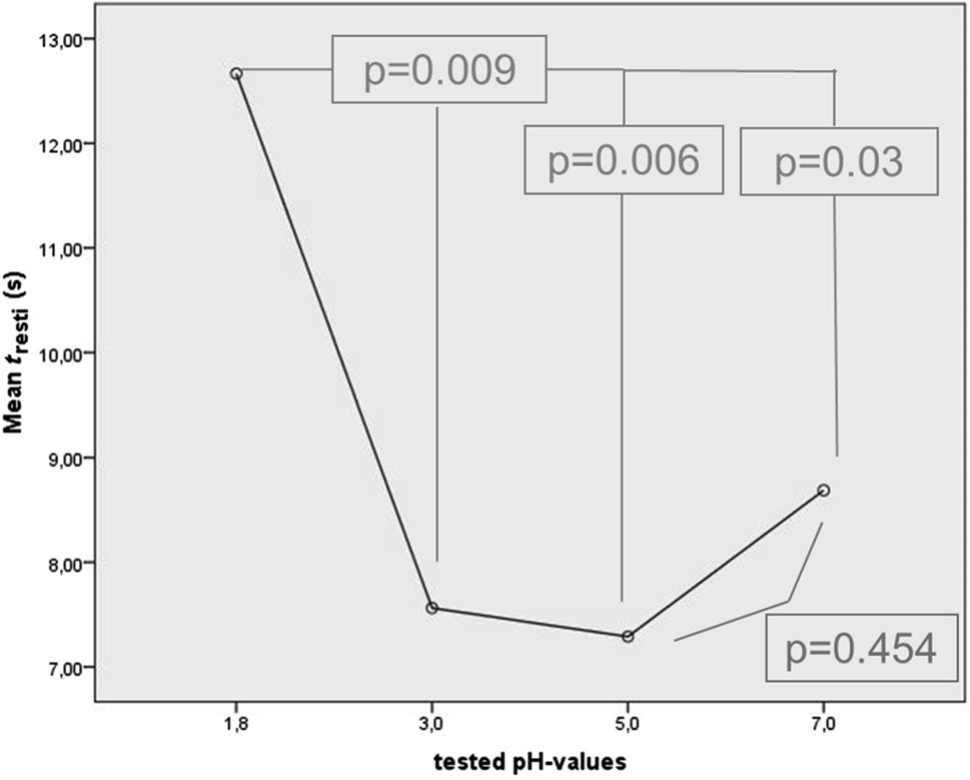
Mean Restitution Times (*t*_resti_) in seconds (*y*-axis) for boli of different pH-consistencies (*x*-axis) with *p-*values for statistical comparisons

Highest mean Restitution Times (Table 1, Fig. 4) occurred in association with swallows of pH 1.8 and measured 12.67 s (SD ± 7.03 s) on average. Mean Restitution Times in relation with boli of pH 7 measured 8.69 s (SD ± 2.72 s), 7.56 s (SD ± 2.23 s) for pH 3 and 7.29 s (SD ± 2.55 s) for pH 5.

**Table 1:** Mean values and standard deviations for the evaluated parameters and different pH-modalities

	pH	*n*	MW	SD
*t*_resti_ (ms)	1.8	10	12.67	7.03
	3	10	7.56	2.29
	5	10	7.29	2.54
	7	10	8.69	2.72
Resting Pressure (mmHg)	1.8	10	58.25	20.52
	3	10	52.48	22.30
	5	10	51.23	21.40
	7	10	62.06	26.68
*t*_relax_ (ms)	1.8	10	0.85	0.35
	3	10	0.73	0.18
	5	10	0.73	0.15
	7	10	0.72	0.13
pre-max (mmHg)	1.8	10	136.47	53.45
	3	10	121.04	64.56
	5	10	116.32	63.12
	7	10	121.74	40.90
post-max (mmHg)	1.8	10	322.47	91.70
	3	10	271.05	41.16
	5	10	254.49	67.59
	7	10	294.02	82.16
*t*_UESact_ (ms)	1.8	10	0.88	0.12
	3	10	0.77	0.11
	5	10	0.77	0.09
	7	10	0.79	0.15
Residual Pressure (mmHg)	1.8	10	− 18.86	5.30
	3	10	− 19.14	4.93
	5	10	− 16.61	5.53
	7	10	− 15.78	7.06

**Fig. 5:**
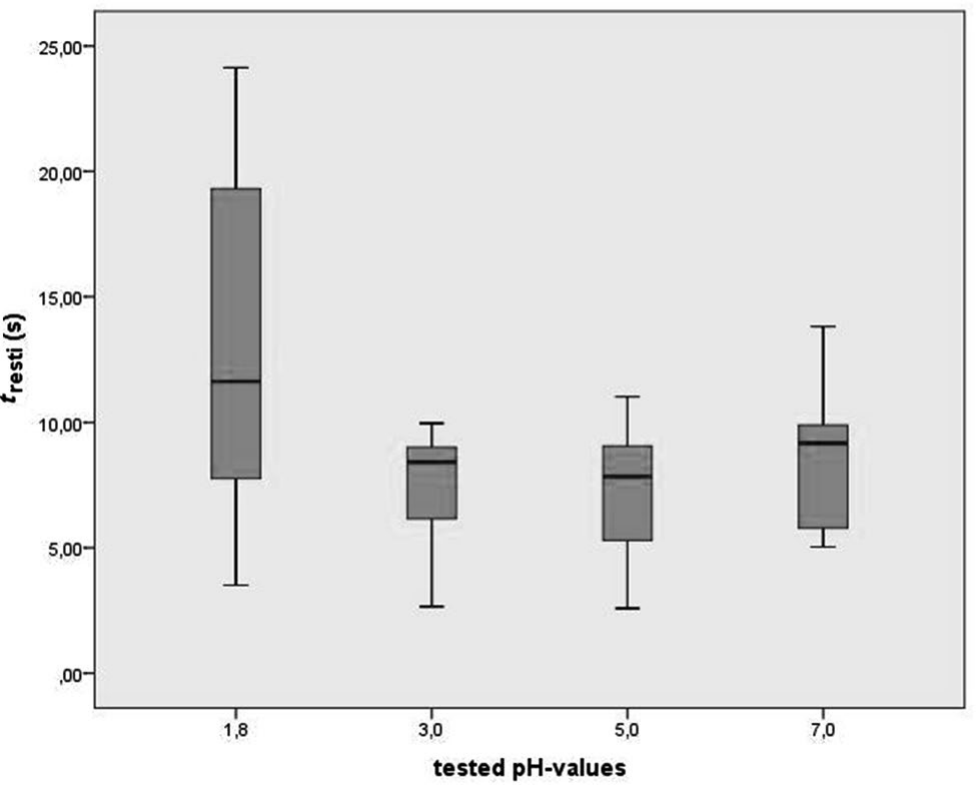
Box plot of Restitution Times (*t*_resti_) in seconds (y-axis) for each pH-value (x-axis)

Mean Restitution Times for swallows with the highest acid concentration of pH 1.8 were significantly higher than in relation with any other pH value (Figs. 4, 5). Differences between pH-neutral consistencies and lower levels of acid concentration did not show statistical significance.

In 40 swallows out of the 400 experimental swallows, no restitution had occurred—i.e., the previously measured Resting Pressure has not been re-reached—before the following swallow took place. 33 out of these were swallows of a bolus with a pH-concentration of 1.8, three in relation with pH 3, two in relation with pH 5 and two with neutral swallows.

**Fig. 6:**
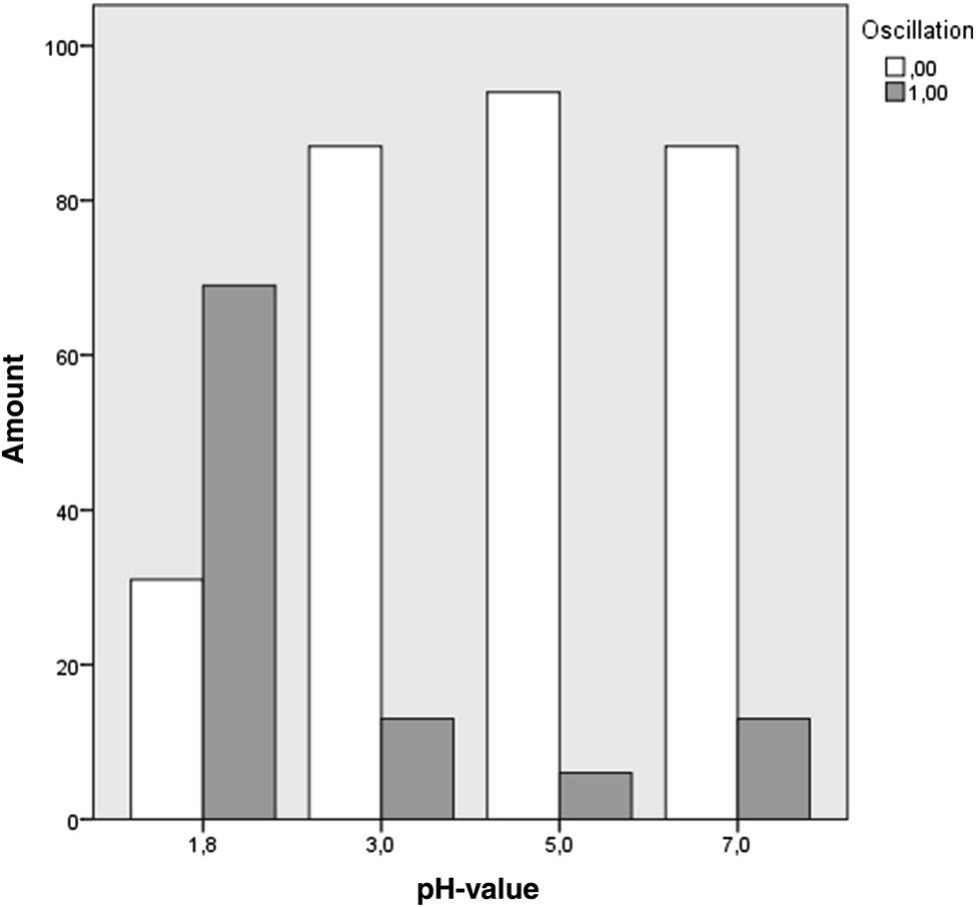
Distribution of Oscillations (*y*-axis) across pH-concentrations (*x*-axis). White marks swallows where no Oscillation occurred and gray the swallows that showed Oscillations

101 Oscillations occurred in 400 swallows. Oscillations were found in 69% of the swallows of pH 1.8, 13% of swallows in relation with pH 3, 6% of pH 5 swallows and 13% of pH 7 swallows (Fig. 6). Binomial testing for each pH-concentration revealed high statistical significance between both groups (*p* < 0.001, Oscillations yes/no). Cross-classified table comparisons and Paersons’s Chi-Square Test comparisons of oscillation frequency distributions revealed that Oscillations occurred significantly more often in relation with swallows of pH 1.8 boli than any other pH-concentration (*p* < 0.001). Comparisons among swallows of pH-concentrations other than 1.8 did not show statistical significance. As for acid swallows (pH 1.8), 9 out of 10 subjects showed Oscillations. Across the 10 swallows, four subjects showed Oscillations with every swallow. The one subject that did not show Oscillations in relation with pH 1.8 did also not show Oscillations with any other level of acidity. The four subjects showing Oscillations with every single swallow in relation with pH 1.8 also did show Oscillations with other pH-consistencies, although much less frequently.

### Resting Pressure

Mean Resting Pressures of all subjects were as high as 62.16 mmHg (SD ± 24.67) for the control swallows of pH 7, 58.28 mmHg (SD ± 20.52 mmHg) for swallows of the strongest acidity (pH 1.8), 52.48 mmHg (SD ± 22.30 mmHg) for pH 3 and 51.23 mmHg (SD ± 21.40 mmHg) for pH 5 accordingly (Table 1). Differences between mean values of swallows of different bolus acidities did not reach statistical significance (Table 2).

**Table 2:** Multiple comparisons of the evaluated parameters

Post-Hoc	pH (I)	pH (J)	Mean difference (I–J)	Significance
*t*_resti_ (ms)	1.8	3	5.10	0.009
		5	5.378	0.006
		7	4.0	0.038
	3	1.8	− 5.10	0.009
		5	0.27	0.883
		7	− 1.12	0.546
	5	1.8	− 5.38	0.006
		3	− 0.27	0.883
		7	− 1.40	0.454
	7	1.8	− 4.0	0.038
		3	1.12	0.546
		5	1.40	0.454
Resting Pressure (mmHg)	1.8	3	5.80	0.564
		5	7.05	0.484
		7	− 3.78	0.707
	3	1.8	− 5.80	0.564
		5	1.25	0.901
		7	− 9.58	0.343
	5	1.8	− 7.05	0.484
		3	− 1.25	0.901
		7	− 10.83	0.284
	7	1.8	3.78	0.707
		3	9.58	0.343
		5	10.83	0.284
*t*_relax_(ms)	1.8	3	0.12	0.199
		5	0.13	0.192
		7	0.13	0.178
	3	1.8	− 0.12	0.199
		5	0.00	0.983
		7	0.01	0.948
	5	1.8	− 0.13	0.192
		3	− 0.00	0.983
		7	0.00	0.965
	7	1.8	− 0.13	0.178
		3	− 0.01	0.948
		5	− 0.00	0.965
pre-max (mmHg)	1.8	3	15.43	0.544
		5	20.15	0.429
		7	14.73	0.562
	3	1.8	− 15.43	0.544
		5	4.72	0.852
		7	− 0.70	0.978
	5	1.8	− 20.15	0.429
		3	− 4.72	0.852
		7	− 5.42	0.831
	7	1.8	− 14.73	0.562
		3	0.70	0.978
		5	5.42	0.831
post-max (mmHg)	1.8	3	51.42	0.125
		5	67.98	0.045
		7	28.45	0.390
	3	1.8	− 51.42	0.125
		5	16.56	0.616
		7	− 22.97	0.487
	5	1.8	− 67.98	0.045
		3	− 16.56	0.616
		7	− 39.53	0.235
	7	1.8	− 28.45	0.390
		3	22.97	0.487
		5	39.53	0.235
Residual Pressure (mmHg)	1.8	3	0.28	0.914
		5	− 2.25	0.388
		7	− 3.08	0.240
	3	1.8	− 0.28	0.914
		5	− 2.53	0.333
		7	− 3.36	0.200
	5	1.8	2.25	0.388
		3	2.53	0.333
		7	− 0.83	0.749
	7	1.8	3.08	0.240
		3	3.36	0.200
		5	0.83	0.749
*t*_UESact_ (ms)	1.8	3	0.11	0.050
		5	0.11	0.042
		7	0.09	0.120
	3	1.8	− 0.11	0.050
		5	0.00	0.936
		7	0.02	0.662
	5	1.8	− 0.11	0.042
		3	− 0.00	0.936
		7	− 0.03	0.605
	7	1.8	− 0.09	0.120
		3	− 0.02	0.662
		5	0.03	0.605

### Residual Pressure

Residual Pressures (Table 1) of as low as − 15.78 mmHg (SD ± 7.06 mmHg) resulted for consistencies of pH 7, − 18.86 mmHg (SD ± 5.30 mmHg) for pH 1.8, − 19.14 mmHg (SD ± 4.93 mmHg) for pH 3, − 16.61 mmHg (SD ± 5.53 mmHg) for pH 5. No significant differences between mean values of swallows of different bolus acidities were seen (Table 2).

### Maximal Values

#### Pre-Swallow Maximum (pre-max)

Mean pre-max values (Table 1) measured as high as 121.74 mmHg (SD ± 40.90 mmHg) for consistencies of pH 7, 136.47 mmHg (SD ± 53.45 mmHg) for pH 1.8, 121.04 mmHg (SD ± 64.56 mmHg) for pH 3 and 116.32 mmHg (SD ± 63.12 mmHg) for pH 5. Means do differ largely between the swallows of highest acidity and all other investigated acidities, but no statistically significant differences were found (Table 2).

#### Post-Swallow Maximum (post-max)

Mean post-max values (Table 1) measured as high as 294.02 mmHg (SD ± 82.16 mmHg) for consistencies of pH 7, 322.47 mmHg (SD ± 91.71 mmHg) for pH 1.8, 271.05 mmHg (SD ± 41.12 mmHg) for pH 3, 254.49 mmHg (SD ± 67.59 mmHg) for pH 5. Mean values differed largely between the highest acidity of pH 1.8 and the others (pH 7, 5 and 3), but only the pairwise comparison between pH 1.8 and pH 5 reached statistical significance (Table 2).

### Time Intervals

#### Relaxation Time (*t*_relax_)

Longest Mean Relaxation Times of 0.854 s (SD ± 0.351 s) occurred with boli of pH 1.8, followed by 0.730 s (SD ± 0.118 s) for pH 3, 0.728 s (SD ± 0.153 s) for pH 5 and 0.724 s (SD ± 0.133 s) for pH 7 (Table 1). Mean values between the highest bolus acidity (1.8) and pH 3 and 5 as well as the pH-neutral swallows differed largely, but did not reach statistical significance (Table 2).

#### Period of Sphincter Activity (*t*_UESact_)

Mean activities in the UES of 0.881 s (SD ± 0.122 s) were longest for pH-values of 1.8, compared to 0.795 s (SD ± 0.155 s) for pH 7, 0.771 s (SD ± 0.109 s) for pH 3 and 0.767 s (SD ± 0.880 s) for pH 5 (Table 1). Mean activity times in the UES differ greatly between the highest acid of pH 1.8 and boli of lower acidity, respectively, pH-neutral boli. Only the pairwise comparisons between pH 1.8 and 3, as well as 5, showed statistical significance (Table 2).

## Discussion

This study investigated the effects a swallowed acid bolus has on UES functions. As the results of this study show, swallowing parameters are influenced by the exposure to extreme levels of swallowed acidity (Table 3).

**Table 3:** Distribution of Oscillations across subjects and pH-values

Subject	pH 1.8	pH 3	pH 5	pH 7
1	0	0	0	0
2	8	0	0	0
3	5	0	0	0
4	6	0	0	0
5	10	0	2	1
6	3	0	0	0
7	10	6	1	1
8	10	1	0	0
9	10	6	3	9
10	7	0	0	2
Total	69	13	6	13

Results suggest that acid exposure results in more effortful swallowing activity, reflected on the one hand by higher maximum pressures (post-max) and on the other hand by prolonged activity times (*t*_resti_, *t*_UESact_) in relation with the most acidic bolus, which most probably act to enhance acid clearance. In general, two phenomena or theories can be put forward: Acid clearance is supported by more muscle force during swallowing (i.e., effortful swallowing activity), as well as extending the time frame of the swallow and the activation times of individual structures.

The primary parameter Restitution Time as well as Oscillations of the UES were able to capture the phenomenon triggered by acid swallows quite well. Whereas pH-neutral swallows showed similar patterns of restitution in the UES and looked like the example swallow in Fig. 3a [26, 38], acid swallows often showed extremely prolonged Restitution Times with persistent very high UES pressures (referred to as Oscillations), as demonstrated in Fig. 3b. Apart from showing those muscle spasms, strong acidity was also often linked to repeated swallows. In some cases an apparent restitution to Resting Pressures occurred, which was then followed by reoccurring periods of very high pressures. The difference between swallows of a pH-neutral bolus to a bolus of strong acidity (pH 1.8) is evident and seems to represent a mechanism of the sphincter to cope with strong acidities by either trying to continuously start the process of clearance and/or by raised muscle tension in order to close the passage into or back out of the esophagus (reflux) in order to protect vital structures. This reaction could not clearly be identified in swallows of a less acidic bolus (pH 3, pH 5), representing acid levels we encounter during regular food intake. These swallows were not obviously distinguishable from neutral swallows and apparently do not trigger a protective mechanism in the same way an extremely sour bolus does. It can hence be concluded that the prolonged Restitution Time does serve a protective function. When the mucosa of the pharynx and/or UES is exposed to the extreme stimulus of high acidity, it appears that a reflexive reaction is triggered, by sending signals—presumably via the afferent fibers of the glossopharyngeal or vagus nerve—to the central nervous system (CNS) in order to initiate a pressure increase of the closing muscles of the UES.

The postulated, but not confirmed, protective function of the UES is a pressure increase during reflux events in order to prevent pharyngolaryngeal reflux (PLR) and concomitant aspiration of gastric fluid. The reactions seen in this study might suggest that pharyngeal exposure to extreme acids triggers a reflexive pressure increase and spasm of the UES. This reaction is most likely caused by exposure of the pharyngeal mucosa to extreme acidity. Hence, receptors suggested to initiate the reflex bow are postulated to exist in the esophagus [29], on the bases of this investigation most likely (also) exist in the pharynx. As it is known that apart from the tongue taste buds also exist on the mucosa of the palate, pharyngeal wall and even the epiglottis [30], it would be possible that these cells are responsible for the detection of a sour bolus, too. Furthermore, the work group around Kummer has reported about the existence of brush cells able to detect a bitter taste throughout the body [31], including the gastrointestinal tract [32] and airways (nose, transition to pharynx, trachea, etc.) [33]. The muscle spasm seen in the UES was suggested to be caused as a reflexive protective mechanism. Whether this activity was already triggered in the pharynx, or only after passing through the UES, however, cannot clearly be answered from this study design. Further studies will need to investigate this postulate in the future as due to the nature of the swallowing path, no clear distinction of receptor location could be made and should also clarify if even oral receptors trigger UES reactions. As (for muscle activity reasons) during this study the bolus was not held in the oral cavity, but transferred into the oral cavity and swallowed immediately on the investigator’s command, no statement can be made on this resulting question.

As for the reaction of the UES in association with acid, Hunt et al. [34] have suggested that raised resting pressures might occur in relation with reflux esophagitis and/or lower esophageal disorders. Brady and colleagues [35] suggested that a premature contraction of the UES during swallowing might be related to the presence of gastroesophageal reflux (GER). Furthermore, the authors hypothesize that further changes should be related, such as a delay in relaxation as well as incomplete relaxation. Subjects of this study, however, did not report of any symptoms in relation with GER. The related changes could not be seen during this study. In fact, if at all, Relaxation Times seem to have been slightly prolonged in relation with acidity.

## Conclusion

Prolonged Restitution Times may represent a reflexive protective mechanism triggered by receptors in the pharyngeal mucosa or the UES preventing regurgitation of acid into the pharynx and larynx, besides representing ongoing attempts of acid clearance. Exposure to high levels of acidity by a swallowed bolus does influence UES functions during swallowing.

## Limitations

Apart from Restitution Times, the parameters post-max and *t*_UESact_ also showed significant effects towards the strongest acid. Due to planning of the study these significances do not qualify as confirmation, but they do imply a rather large effect of the acid and should be verified in a follow-up study. Whether the other parameters really were not influenced by acidity cannot be concluded finally, as the sample size of this study—due to the rather large effect found in Restitution Times—might have masked smaller effects. Furthermore, the relatively large standard deviations in this study made it hard to show small effects. The effect of prolonged Restitution Times is therefore even more remarkable. Other (strong) trends seen in this study might need a larger test population to be confirmed. Furthermore, by aggregating the swallowing values per person and modality the degrees of freedom were reduced. As a result of this reduction tests do not show significances easily. Mean value comparisons, however, did show strong tendencies for further parameters such as pre-max.

As has previously been described by other authors [36, 37], with many of the parameters a continuing adjustment to the catheter could be found. This effect caused the first values, which in this study were baseline swallows in relation with a bolus of pH 7, to often be higher than the same parameter at a later point in time during the study. A randomization of the bolus acidity order would certainly have helped to eliminate this effect, but the small number of cases necessary to prove the effect for Restitution Times did not allow for sufficient combinations.

As described above, values cannot easily be compared to values of other studies in this field of research, as catheters, bolus volumina and experimental setups differ greatly. Most studies investigated the UES in relation with acid exposure to the esophagus, generally by infusion or spontaneous reflux events, whereas during this study acid was swallowed. Moreover, most of the studies investigating acid exposure used water-perfused or stationary pull through manometry rather than solid-state catheters. Due to the ‘constant’ perfusion of liquid in water-perfused manometry, and the resulting primary or secondary ‘swallowing’ activity, the resulting values can only be compared to a limited extend.

## Outlook

Analyzing the results of this study, it was not possible to localize—if existent—receptors initiating the reflex bow activity for increased UES pressures when exposed to extreme acidity. In a following study, the infusion of minimal amounts of acid into the pharynx without addressing esophageal receptors shall clarify whether or not pharyngeal receptors sensitive to pH consistencies exist to trigger reflex bow activities.

It might also be interesting to investigate in a different study whether the swallow-associated behavior of the UES, Restitution Times and Oscillations, in particular, do differ from pH-neutral swallows if no pH 1.8 boli were swallowed previously, as the structures involved might already be ‘used’ to acid by the previous exposure to extreme acidity and therefore show a different reaction.
