# Correction to: Influence of Acid Swallows on the Dynamics of the Upper Esophageal Sphincter

**DOI:** 10.1007/s00455-020-10210-2

**Published:** 2020-11-13

**Authors:** Simone Miller, Martin Ptok, Michael Jungheim

**Affiliations:** Department of Phoniatrics and Pediatric Audiology, Hannover Medical School, Carl-Neuberg-Straße 1, Hannover, Germany

## Correction to: Dysphagia 10.1007/s00455-020-10159-2

The article “**Influence of Acid Swallows on the Dynamics of the Upper Esophageal Sphincter”**, written by “**Simone Miller, Martin Ptok, Michael Jungheim**”, was originally published electronically on the publisher’s internet portal on 4 September 2020 without open access. With the author(s)’ decision to opt for Open Choice the copyright of the article changed on 30 October 2020 to © The Author(s) 2020 and the article is forthwith distributed under a Creative Commons Attribution 4.0 International License (https://creativecommons.org/licenses/by/4.0/), which permits use, sharing, adaptation, distribution and reproduction in any medium or format, as long as you give appropriate credit to the original author(s) and the source, provide a link to the Creative Commons licence, and indicate if changes were made.

The images or other third party material in this article are included in the article’s Creative Commons licence, unless indicated otherwise in a credit line to the material. If material is not included in the article’s Creative Commons licence and your intended use is not permitted by statutory regulation or exceeds the permitted use, you will need to obtain permission directly from the copyright holder. To view a copy of this licence, visit http://creativecommons.org/licenses/by/4.0.

The original article has been corrected.

